# Rationale for WHO's New Position Calling for Prompt Reporting and Public Disclosure of Interventional Clinical Trial Results

**DOI:** 10.1371/journal.pmed.1001819

**Published:** 2015-04-14

**Authors:** Vasee S. Moorthy, Ghassan Karam, Kirsten S. Vannice, Marie-Paule Kieny

**Affiliations:** World Health Organization, Geneva, Switzerland

## Abstract

Vasee Moorthy and colleagues explain why the WHO is calling for public disclosure of clinical trial results.

On April 14, 2015, the World Health Organization (WHO) published a new statement on the public disclosure of clinical trial results (S1 Text) [1]. The WHO statement not only re-affirms the ethical imperative of clinical trial results reporting, it also defines reporting timeframes, calls for results-reporting of older but still unpublished trials, and outlines steps to improve linkages between clinical trial registry entries and their published results. This updates and expands WHO’s 2005 statement that “the registration of all interventional trials is a scientific, ethical, and moral responsibility” [2].

WHO’s 2005 statement called for all interventional clinical trials to be registered. Subsequently, there has been an increase in clinical trial registration prior to the start of trials. This has enabled tracking of the completion and timeliness of clinical trial reporting. There is now a strong body of evidence showing failure to comply with results-reporting requirements across intervention classes, even in the case of large, randomised trials [3–7]. This applies to both industry and investigator-driven trials. In a study that analysed reporting from large clinical trials (over 500 participants) registered on clinicaltrials.gov and completed by 2009, 23% had no results reported even after a median of 60 months following trial completion; unpublished trials included nearly 300,000 participants [3]. Among randomised clinical trials (RCTs) of vaccines against five diseases registered in a variety of databases between 2006–2012, only 29% had been published in a peer-reviewed journal by 24 months following study completion [4]. At 48 months after completion, 18% of trials were not reported at all, which included over 24,000 participants. In another study, among 400 randomly selected clinical trials, nearly 30% did not publish the primary outcomes in a journal or post results to a clinical trial registry within four years of completion [5].

The Declaration of Helsinki and other statements have outlined the compelling reasons why interventional clinical trials should be reported in a timely fashion [8–10]. In brief, not reporting clinical trial results is likely to lead to dissemination bias. This bias has the following major adverse consequences:

- It affects understanding of the scientific state of the art.
- It leads to inefficiencies in resource allocation for both research and development and financing of health interventions.
- It creates indirect costs for public and private entities, including patients themselves, who pay for suboptimal or harmful treatments.
- It potentially distorts regulatory and public health decision making.

Furthermore, it is unethical to conduct human research without publication and dissemination of the results of that research. In particular, withholding results may subject future volunteers to unnecessary risk.

The realisation of the importance of reporting results from clinical trials has led to the introduction of reporting requirements in an increasing number of jurisdictions. Requirements have been articulated in the current Declaration of Helsinki [8], by legislation in the United States [11], and at the European Union level [12]. The International Committee of Medical Journal Editors encourages posting of clinical trial results in clinical trial registries [13]. It has recently become mandatory for sponsors of clinical trials registered with the EU Clinical Trials Register to post results summaries on the registry database [12]. The US Department of Health and Human Services has proposed an expanded scope for clinical trials to be reported by law and clarified reporting requirements for posting results on clinicaltrials.gov. In addition, the US National Institutes of Health (NIH) is proposing to mandate clinical trials registration and results reporting for all NIH-funded clinical trials [14]. Initiatives such as Cochrane, AllTrials, and the OPEN Consortium (To Overcome failure to Publish nEgative fiNdings) are advocating strongly for greater transparency in results reporting. During 2014, one of the world’s largest pharmaceutical companies adopted policies for public disclosure of clinical research that include posting of results from all clinical trials (Phase I–IV) on a free-to-access website and submission to a peer-reviewed journal in a short timeframe, going far beyond what was required by laws and regulations [15]. Despite the recent progress in some jurisdictions, gaps remain.

In its statement, WHO has outlined the need for reporting to occur in two modalities. The first is for the main findings of clinical trials to be submitted for publication in a peer-reviewed journal within 12 months of study completion (defined as the final data collection date for the primary outcome measure), with a further 12 months allowed from first submission to publication. Thus the key indicator for tracking will be journal publication of results within 24 months of study completion. Additionally, the key outcomes (defined in the statement) should be made publicly available within 12 months of study completion by posting to the results section of the primary clinical trial registry. If the registry does not allow posting, then the results should be posted on another easily accessible website.

Another important feature of the WHO statement is its call for public disclosure of results from older, unreported clinical trials. Results from past clinical trials still have an important bearing on scientific research today. Additional components of the WHO statement are a re-affirmation of registration of clinical trials before the first patient receives the medical intervention, and inclusion of a Trial ID (i.e., the clinical trial registry identifier) in the publication for easy linking of manuscripts with clinical trial registry entries. This will assist the ready identification of trials that have been conducted but not reported. It may be appropriate for funding agencies, particularly those spending taxpayer-sourced funds, to engage in such tracking as part of routine auditing for good use of research funds.

In 2005, WHO established the International Clinical Trials Registry Platform (ICTRP) to provide an overview of clinical trial research accessible to all those involved in health care decision making globally [2]. The ICTRP is a registry platform that regularly imports trial records from clinicaltrials.gov, ISRCTN, EU Clinical Trials Register, Australia New Zealand Clinical Trial Registry, Pan African Clinical Trial Registry, and Clinical Trial Registries from China, India, Brazil, Republic of Korea, Cuba, Germany, Iran, Japan, Sri Lanka, The Netherlands, and Thailand.

A current obstacle to reporting results is the lack of mechanisms on many clinical trial registries for sponsors to submit results for public access. Clinicaltrials.gov and the EU Clinical Trials Register are two registries that do so.

Ensuring that all interventional clinical trials are reported will require action following the WHO statement. Data indicate that the extent of results-reporting has been insufficient to date and that incentives and legislation are needed to achieve compliance. WHO calls for ethics committees, regulatory authorities, professional bodies, sponsors, investigators, and funding agencies to act in their jurisdictions to ensure results from all interventional clinical trials are reported and publicly disclosed.

## Supporting Information

S1 Text WHO statement on public disclosure of clinical trial results. (PDF)
