# Supplementary material for: Rationale for WHO's New Position Calling for Prompt Reporting and Public Disclosure of Interventional Clinical Trial Results
Source: PLoS Med. 2015 Apr 14;12(4):e1001819. doi: 10.1371/journal.pmed.1001819 (PMC4396122; doi:10.1371/journal.pmed.1001819)
Supplement: S1 Text — (PDF) [file pmed.1001819.s001.pdf]

WHO Statement on Public Disclosure of Clinical Trial Results

Background

Following a ministerial summit on Health Research in 2004, a World Health Assembly Resolution passed in 2005 called for unambiguous identification of all interventional clinical trials. This led to the establishment of the WHO International Clinical Trials Registry Platform, which collates information on trials that have been notified in a network of clinical trial registries (who.int/ictrp/network). WHO's existing position on registration is available at who.int/ictrp: "The registration of all interventional trials is a scientific, ethical and moral responsibility". Deposition of information on trials in such registries, prior to their initiation, is a condition for publishing the results of trials in many leading medical journals[1]. However, concerns have been raised that there may be selective publication of trials dependent on their results, with particular concern that trial results which may be viewed as "negative", are less likely to be submitted, or accepted, for publication in the scientific literature or made public in other ways. Notification of trials to clinical trial registries has become more widespread, and it is possible to evaluate what proportions of recorded trials have not reported results at different times after the planned end dates of the trials. Multiple analyses have confirmed that a substantial number of clinical trials remain unreported several years after study completion, even in the case of large randomized clinical trials.

In the latest version of the Declaration of Helsinki it is stated that "Every research study involving human subjects must be registered in a publicly accessible database before recruitment of the first subject." and that "Researchers have a duty to make publicly available the results of their research .... Negative and inconclusive as well as positive results must be published or otherwise made publicly available". There is an ethical imperative to report the results of all clinical trials, including those of unreported trials conducted in the past. Furthermore poor allocation of resources for product development and financing of available interventions, and suboptimal regulatory and public health recommendations may occur where decisions are based on only a subset of all completed clinical trials.

Reiteration of WHO position on clinical trial registry sites

Before any clinical trial[2] is initiated (at any Phase) its details are to be registered in a publicly available, free to access, searchable clinical trial registry complying with WHO's international agreed standards. The clinical trial registry entry should be made before the first subject receives the first medical intervention in the trial.

---

[1] www.icmje.org/recommendations/browse/publishing-and-editorial-issues/clinical-trial-registration.html

[2] A clinical trial is defined as any experimental study which prospectively allocates humans to a medical intervention. While randomised assignments are considered of highest value for assessing the safety and efficacy of an intervention, the WHO definition of a clinical trial for reporting purposes also includes non-randomised assignments, for example in Phase I trials. As safety problems may occur in Phase I trials, it is critical that the same disclosure mechanisms apply for non-randomised Phase I trials as apply to randomised Phase I, II or III trials. Phase IV or post-licensure trials of health products are considered clinical trials if they involve prospective designs (with or without randomisation). Clinical trials have pre-specified research or product development objectives, and so routine use of health interventions without specified research objectives are not considered to be within the definition of a clinical trial.

Updating clinical trial registry entries

All clinical trial registry sites are to be updated as necessary to include final enrolment numbers achieved, and the date of actual study completion (defined as the last data collection timepoint for the last subject for the primary outcome measure). If clinical trials are terminated, their status is to be updated to note the termination, and to report the numbers enrolled up to the point of termination.

Reporting timeframes for clinical trials

Clinical trial results are to be reported according to the timeframes outlined below. Reporting is to occur in BOTH of the following two modalities.

1. The main findings of clinical trials[3] are to be submitted for publication in a peer reviewed journal within 12 months of study completion[4] and are to be published through an open access mechanism unless there is a specific reason why open access cannot be used, or otherwise made available publicly at most within 24 months of study completion.
2. In addition, the key outcomes[5] are to be made publicly available within 12 months of study completion[4] by posting to the results section of the primary clinical trial registry. Where a registry is used without a results database available, the results should be posted on a free-to-access, publicly available, searchable institutional website of the Regulatory Sponsor, Funder or Principal Investigator.

It is noted that several journals allow open access publication of clinical trial findings. Some journals have an explicit policy of supporting publication of negative trials. These 12 month and 24 month timeframes represent the longest possible acceptable timeframe for reporting and shorter timeframes are strongly encouraged. It should be possible in most instances for reporting to occur in shorter timeframes.

Reporting of past clinical trials results

Unreported clinical trials conducted in the past are to be disclosed in a publicly available, free to access, searchable clinical trial registry. In addition it is desirable that unreported clinical trials are published in a peer reviewed journal.

Inclusion of Trial ID in clinical trial publication

The Trial ID or registry identifier code/number is always to be included in all publications of clinical trials, and should be provided as part of the abstract to PubMed and other bibliographic search databases for easy linking of trial reports with clinical trial registry site records. Bibliographic search

---

[3] See www.consort-statement.org for broadly accepted standards on presentation of results in peer reviewed manuscripts reporting clinical trials

[4] Defined as the last data collection timepoint for the last subject for the primary outcome measure

[5] "Key outcomes" here is defined as including the following as a minimum: participant flow, baseline characteristics, primary and secondary outcome measures, and adverse events including all serious adverse events and important anticipated or unanticipated adverse events. An example of a format for providing results: https://clinicaltrials.gov/ct2/about-site/results. Note that "key outcomes" refers to analyses conducted on data, not to primary data disclosure itself.

databases such as PubMed are encouraged to make Trial IDs easily available by inclusion in the abstract of each clinical trial record.

Note on Data Sharing Initiatives

The benefit of sharing research data and the facilitation of research through greater access to primary datasets is a principle which WHO sees as important. This statement is not directed towards sharing of primary data. However WHO is actively engaged with multiple initiatives related to data sharing, and supports sharing of health research datasets whenever appropriate. WHO will continue to engage with partners in support of an enabling environment to allow data sharing to maximise the value of health research data.
